# Xanthine Oxidoreductase-Derived Reactive Species: Physiological and Pathological Effects

**DOI:** 10.1155/2016/3527579

**Published:** 2015-12-28

**Authors:** Maria Giulia Battelli, Letizia Polito, Massimo Bortolotti, Andrea Bolognesi

**Affiliations:** Alma Mater Studiorum-University of Bologna, Department of Experimental, Diagnostic and Specialty Medicine (DIMES), General Pathology Unit, Via S. Giacomo 14, 40126 Bologna, Italy

## Abstract

Xanthine oxidoreductase (XOR) is the enzyme that catalyzes the oxidation of hypoxanthine to xanthine and xanthine to uric acid and is widely distributed among species. In addition to this housekeeping function, mammalian XOR is a physiological source of superoxide ion, hydrogen peroxide, and nitric oxide, which can function as second messengers in the activation of various pathways. This review intends to address the physiological and pathological roles of XOR-derived oxidant molecules. The cytocidal action of XOR products has been claimed in relation to tissue damage, in particular damage induced by hypoxia and ischemia. Attempts to exploit this activity to eliminate unwanted cells via the construction of conjugates have also been reported. Moreover, different aspects of XOR activity related to phlogosis, endothelial activation, leukocyte activation, and vascular tone regulation, have been taken into consideration. Finally, the positive and negative outcomes concerning cancer pathology have been analyzed because XOR products may induce mutagenesis, cell proliferation, and tumor progression, but they are also associated with apoptosis and cell differentiation. In conclusion, XOR activity generates free radicals and other oxidant reactive species that may result in either harmful or beneficial outcomes.

## 1. Introduction

The enzyme xanthine oxidoreductase (XOR) has a wide distribution throughout living organisms and is highly conserved in prokaryotic, plant, and animal species (reviewed in [1]). XOR is a dimeric metalloflavoprotein comprising two identical subunits of approximately 145 kDa each, including one molybdenum-containing molybdopterin cofactor (Mo-co) and one flavin adenine dinucleotide (FAD) cofactor, as well as two nonidentical iron-sulfur redox centers. The purine oxidation occurs at the Mo-co site, while the FAD site is the oxidized nicotinamide adenine dinucleotide (NAD^+^) and O_2_ reduction sites. The electron flux moves between the Mo-co and FAD cofactors through the two iron-sulfur clusters (reviewed in [2]).

XOR catalyzes the oxidation of hypoxanthine to xanthine and xanthine to uric acid, which are the last two steps of purine catabolism in the highest primates. XOR has the rate-limiting function of generating irreversible products, thus precluding the salvage pathway of purine nucleotides. Additionally, different endogenous metabolites and various xenobiotics can be oxidized by XOR. Uric acid and its oxidized derivatives may exert prooxidant activity, mainly within the cell; however, it has* in vivo* antioxidant activity, mainly in body fluids. This scavenger action is supposed to provide an evolutionary advantage to primates that lost their uricase activity via mutation and acquired a crucial defense against oncogenesis by free radicals [3].

XOR is highly regulated at both the transcriptional and posttranslational levels. XOR activity is present in all mammalian tissue and fluids, although, in most of them, it is expressed at very low levels because the human XOR gene is usually subjected to a repressing regulation at the transcriptional level [4]. The highest XOR levels are expressed in liver, intestine, kidney, and lactating mammary gland epithelial cells and in vascular endothelial cells (reviewed in [5]). XOR expression may be increased by various stimuli, such as hormones, growth factors, inflammatory cytokines, and low oxygen tension. At the posttranslational level, XOR is modulated with both quantitative and qualitative changes in its activity. XOR protein may be produced in demolybdo- and/or desulfo-forms, which are inactive in xanthine catalysis at the Mo-co site, although they can oxidize the reduced nicotinamide adenine dinucleotide (NADH) at FAD site. These defective XOR forms are present in varying percentages in milk and could be reactivated with the reinsertion of the lacking atoms at the active site. XOR activity was observed to increase in response to hypoxia without changes in the levels of mRNA or enzyme protein, indicating a posttranslational regulation of XOR (reviewed in [6]). However, the most peculiar modulation of XOR activity in mammals consists of the conversion from the dehydrogenase to the oxidase form. This transition occurs in various pathological conditions (reviewed in [7]).

In all organisms, XOR is present in its constitutively active dehydrogenase form, whereas, only in mammals, the NAD^+^-dependent xanthine dehydrogenase (XDH, EC 1.1.1.204) can be converted to the oxidase form (XO, EC 1.1.3.22) through sulfhydryl group oxidation or limited proteolysis [8]. XO delivers electrons directly to molecular oxygen (O_2_), thus generating the reactive oxygen species (ROS), superoxide anion (O_2_^•−^), and hydrogen peroxide (H_2_O_2_), via a one-electron and a two-electron reduction, respectively. This gives rise to the hydroxyl radical (HO^•^) in the presence of iron via the Haber-Weiss and Fenton reactions. The percentage of divalent versus univalent electron transfer to O_2_ and the relative quantities of O_2_^•−^ and H_2_O_2_ generated by XO are dependent upon O_2_ tension, pH, and purine concentration. Thus, under normal physiological conditions, H_2_O_2_ is the major reactive product derived from the XO-catalyzed O_2_ reduction. H_2_O_2_ formation is further favored when both the O_2_ levels and pH are reduced, such as under ischemic and/or hypoxic conditions (reviewed in [9]). Under hypoxic conditions, these ROS can also be produced by XDH, which, at the FAD site, can oxidize NADH. Hypoxia-mediated acidic pH and low O_2_ tension lessen the nitric oxide (NO) formation by NO synthase and increase its potential to uncouple and produce O_2_^•−^. These conditions reduce XOR affinity for xanthine while increasing affinity for nitrites, which compete with xanthine at the Mo-co site and can be reduced to NO. Under the same conditions the amount of O_2_^•−^ formation by XOR is sufficient to react with NO and generate reactive nitrogen species (RNS), particularly peroxynitrite (ONOO^−^). Both free radicals, such as O_2_^•−^, HO^•^, and NO, and nonradical forms, such as H_2_O_2_ and ONOO^−^, have an oxidizing effect, thereby contributing to oxidative stress (reviewed in [10]).

The generation of these oxidants may be only partially blocked by allopurinol, which inhibits the Mo-co site in a competitive manner but does not inhibit the catalytic activity at the FAD site. All together, these products are responsible for XOR cytotoxic and proinflammatory activities and for pro- and antitumorigenic effects, in both physiological and pathological conditions. The various XOR functions are dependent on (i) the level of ROS production, as in the case of cytotoxic effects; (ii) the type of the prevalent product, for instance, NO in the presence of high nitrate level; (iii) the specificity of different cell types, such as phagocytes in inflammation; (iv) the level of XOR gene expression, in particular in cancer.

## 2. Cytotoxicity of Xanthine Oxidoreductase Products

XOR cytotoxicity received much attention during the second half of last century, together with the circumstances of the conversion from XDH to XO. An elevated XO/XDH activity ratio has been reported in different pathological conditions, which were characterized by tissue damage and cell necrosis. In particular, the XDH to XO shift was observed in a variety of hypoxic/ischemic conditions (reviewed in [6]), including organ transplantation (reviewed in [11]). In such circumstances, any reoxygenation/reperfusion could increase the supply of oxygen for the formation of oxidants, but it was not strictly required. Additionally, the conversion from XDH to XO was not necessary for ROS generation, as discussed above, especially in the presence of low oxygen tension that favors the NADH oxidase activity of XOR. However, the formation of XOR-derived ROS was indicated as the causal agent of the injury or, at least, of the damage amplification, although more than one source of ROS could be implicated (reviewed in [12]).

**Figure 1:**
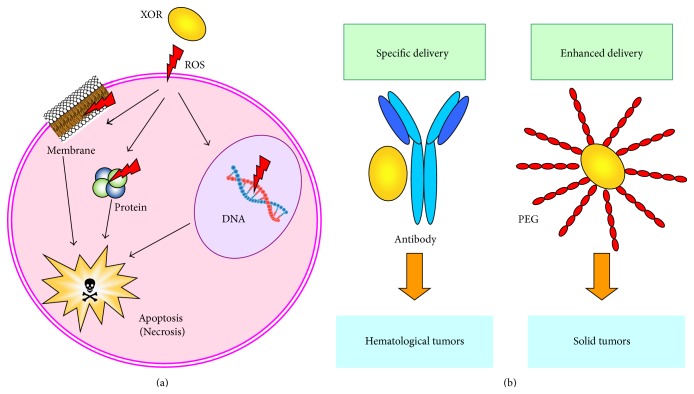
Pharmaceutical applications of xanthine oxidoreductase (XOR) cytotoxicity. (a) Mechanisms of ROS cytotoxicity: ROS induce peroxidation of membrane lipids, DNA damage, and protein oxidation and lead to cell death, mainly via apoptosis through impaired mitochondrial function (reviewed in [13]). (b) XOR was conjugated to carriers for the experimental elimination of specific target cells. Selective cell killing was obtained by conjugating XOR to an antibody that was able to specifically deliver reactive oxygen species (ROS) to target cells [23]. Enhanced ROS delivery to solid tumors was achieved by XOR conjugation to polyethylene glycol (PEG) [24].

The mechanism of ROS cytotoxicity is attributed to peroxidation of membrane lipids, DNA damage, and protein oxidation, which impair mitochondrial function and lead to apoptosis (reviewed in [13]) (Figure 1(a)). Indeed, DNA damage and the consequent loss of cloning efficiency occurred in a Burkitt lymphoma-derived cell line via XOR activity through the production of ROS [14]. Apoptosis and necrosis were induced to proliferating human lymphocytes by XOR-derived oxidative stress, which was prevented by catalase [15]. Additionally, oxidative DNA damage, consequent to the ROS generated by XOR activity, provoked cell death in a nasopharyngeal carcinoma cell line [16]. Accordingly, XOR-derived ROS caused DNA double-strand breaks that were associated with p53 function/expression and caspase-dependent apoptosis in primary human lung microvascular endothelial cells that were exposed to cigarette smoke extract [17].

The oxidative stress could be utilized to eliminate unwanted cells, particularly cancer cells. An attempt to take advantage of the cytotoxicity of XOR products was performed by conjugating the XOR protein to monoclonal antibodies, with the intent of delivering XOR activity to the antigen-bearing cell. XOR-containing conjugates recognizing B lymphocyte antigens were prepared with the purpose of autologous bone marrow grafting. These conjugates selectively killed B lymphoma cell lines [18] without reducing normal myeloid clonogenic efficiency [19] and were effective in bone marrow purging from malignant B lymphocytes [20]. XOR immunotargeting was also studied in an experimental model to eliminate T lymphocytes from bone marrow for heterologous transplantation [21]. The cytotoxicity and selectivity of conjugated XOR were enhanced by the addition of chelated iron that potentiates the free radical formation (reviewed in [22]). The efficacy of XOR activity was proven in conditions that were very similar to the* ex vivo* treatment for bone marrow purging from multiple myeloma cells, with a XOR/antibody conjugate or with a free monoclonal antibody followed by a XOR/anti-antibody conjugate. Both direct and indirect methods induced a prevalence of apoptotic death over necrosis in malignant B lymphocytes [23] (Figure 1(b)).

To improve the ROS delivery efficiency to solid tumors, XOR was conjugated to polyethylene glycol [24], which (i) confers superior* in vivo* pharmacokinetic characteristics by increasing the blood half-life of the enzyme; (ii) counteracts the aspecific adhesiveness of XOR to the vascular inner surface; and (iii) concentrates XOR in cancer tissues by exploiting the enhanced permeability and retention effect of macromolecules and lipids in solid tumors (reviewed in [25]) (Figure 1(b)).

## 3. Proinflammatory Activity of Xanthine Oxidoreductase Products

The evolution of XOR from the highly conserved dehydrogenase to the interconvertible mammalian oxidase form confers to its enzyme activity a new role of producing physiologic signal transduction that is mediated by ROS as secondary messengers (reviewed in [26, 27]).

**Figure 2:**
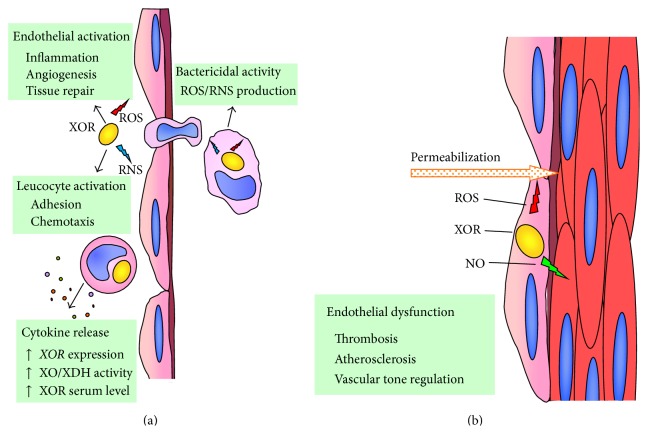
Prophlogistic action of reactive oxygen (ROS) and nitrogen (RNS) species. (a) Interferon and other cytokines increase xanthine oxidoreductase gene (XOR) expression as well as the conversion of xanthine dehydrogenase (XDH) to xanthine oxidase (XO) and XOR serum level (reviewed in [11]). XOR-derived ROS and RNS mediate the endothelial and phagocytic cell activation that is functional in antibacterial defense (reviewed in [30]). (b) XOR products induce endothelial permeabilization and dysregulation of vascular tone, which may lead to thrombosis and atherosclerosis (reviewed in [37]).

XOR activity is known to be upregulated in response to inflammatory cytokines [28], which induce the XDH to XO transition and also increase the XOR level in plasma [11], supporting the hypothesis that XOR is a component of the innate immune system (reviewed in [29]). Indeed, XOR has been implicated in the defense against infectious diseases because of its capability of activating the cellular phlogistic response at various levels (reviewed in [30]). XOR-derived ROS promote leukocyte-endothelial cell interactions by increasing the adhesion of phagocytes [31]. They also induce the production of cytokines [32], thus amplifying the inflammatory response, and chemotactic factors [33], which cause the accumulation of polymorphonuclear granulocytes in the microvasculature [34]. The bactericidal activity of XOR may contribute to the oxygen-dependent cell killing during leukocyte phagocytosis through ROS and ONOO^−^ production [35]. The antibacterial properties of XOR suggest that its abundance in milk could have the role of a natural antibiotic, representing one of the reasons to encourage breastfeeding by mothers [36] (Figure 2(a)).

The usually very low XOR serum level in humans may become more elevated in pathological circumstances that cause tissue damage and the release of XOR from cells into the bloodstream. Circulating XOR is converted to the oxidase form and binds to endothelial cells, even at distant sites, inducing proinflammatory signaling or even remote organ injury (reviewed in [11]). The proinflammatory activity exerted by the XOR-derived ROS may affect the microvascular lining by inducing endothelium permeabilization, which begins both the physiological cascade of immune response and the pathological events that induce atheromatous plaque formation (reviewed in [37]) (Figure 2(b)). The XOR products together with the oxidants generated by NAD(P)H oxidase and NO synthase may also modulate another endothelial cell function, the regulation of arteriolar tone via NO production, which has local and systemic vasodilating activity and causes XOR inhibition (Figure 2(b)). NO is produced by endothelial NO synthase that is inhibited either by ROS or under hypoxic conditions. In these circumstances, NO generation is assured by the nitrite reductase activity of both XOR and NAD(P)H oxidase, which undergo reciprocal activation by generating O_2_^•−^ (reviewed in [38]). As the activities of these enzymes are interdependent in the endothelium, the final outcome is the result of a physiopathological balance amongst their activities. Thus, it is not surprising that both XOR activity and its inhibition by allopurinol may induce endothelial dysfunction and promote platelet aggregation, as well as aggravating hypertension and cardiovascular diseases [39].

In patients with coronary disease, the treatment with the angiotensin receptor blocker losartan reduced the endothelium-bound XOR activity and XOR inhibition with oxypurinol improved endothelium-dependent vasodilation, suggesting that endothelial dysfunction in coronary disease is at least in part dependent on angiotensin II-dependent endothelial XOR activation [40]. In patients with metabolic syndrome, XOR inhibition by allopurinol reduces myeloperoxidase and malondialdehyde blood levels, while increasing the flow-mediated dilation, suggesting that XOR-induced oxidative stress contributes to endothelial vasomotor dysfunction [41]. The underlying mechanism is supposed to be the reduced bioavailability of NO due to the reaction of NO with O_2_^•−^ (reviewed in [39]). However, in grade 1 drug-naïve hypertensive subjects a dietary nitrate load reduces systolic and diastolic blood pressure. This effect is related to an increased NO generation, which is significantly attenuated by allopurinol and is associated with higher levels of erythrocytic XOR expression and nitrite reductase activity in hypertensive patients in comparison to normotensives volunteers [42]. The effects of ROS generated by human XOR on cardiovascular disease have been detailed in two recent publications (reviewed in [11, 37]).

XOR may produce ROS and NO, which are both required for the formation of normal granulation tissue and wound healing. In vitro keratinocytes and endothelial cell proliferation and migration were increased by H_2_O_2_ and nitrite. XOR expression was upregulated shortly after wounding at the wound edge. Locally applied allopurinol, as well as a tungsten-enriched diet that drastically lowered XOR activity, significantly delayed wound healing in mice. The effect was reversed and angiogenesis improved with the topical H_2_O_2_ administration, strongly suggesting that XOR contributes to wound repair [43].

## 4. Pro- and Antitumorigenic Activity of Xanthine Oxidoreductase Products

**Figure 3:**
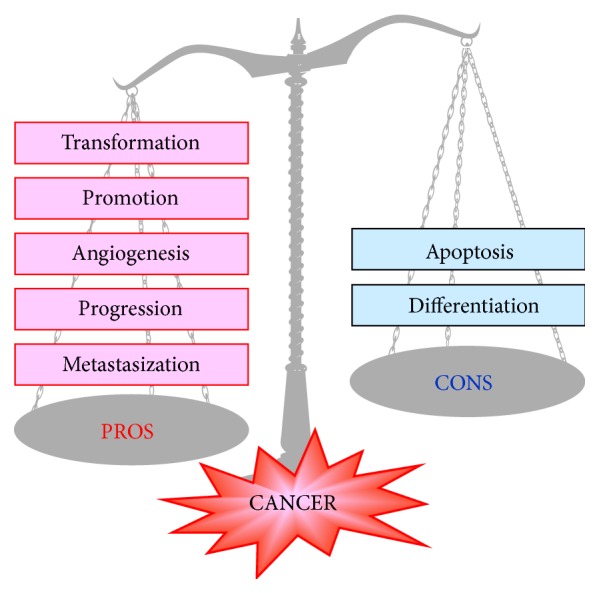
Cancer pathogenesis: ambiguous role of xanthine oxidoreductase (XOR). XOR-derived ROS may activate genes responsible for each phase of cancer development (reviewed in [47]) as well as genes that promote antioncogenic activities (reviewed in [45]).

In both experimental and clinical pathology, the level of XOR expression was often found to be higher or lower in cancer tissues compared with the corresponding normal tissue or to the normal tissues bordering cancerous tissues (reviewed in [44]). In particular, XOR expression and activity in neoplastic human tissues have been recently addressed and discussed together with the XOR role in differentiation and oncogenesis (reviewed in [45]). Moreover, XOR products have been associated with both the process of oncogenesis ([46], reviewed in [47]) and its prevention ([48], reviewed in [49]) (Figure 3).

The level of XOR activity was higher than normal and that of paraoxonase l, a free radical scavenger enzyme, was lower in the serum of patients with various cancer illnesses [50]. A low activity of various oxidative enzymes, in particular XOR, has been reported to correlate with cell proliferation in different settings, including cancer, and a hypothesis has been formulated that a low level of free radicals may stimulate cancer cell growth [51]. XOR can also confer a cancer-promoting action through the above-discussed proinflammatory activities of ROS and RNS.

The analysis of a vast cohort of women followed for 11 years showed a dose-dependent risk of breast cancer by alcohol consumption [52] and a mechanism involving XOR-derived ROS has been proposed for the pathogenesis of this cancer. XDH is expressed at high levels by mammary epithelium, particularly in relation to lactation, and can produce ROS by oxidizing ethanol [53] as well as acetaldehyde and NADH, which are generated by alcohol catabolism. ROS can be responsible for DNA damage, mutagenesis, and neoplastic transformation, especially in aged breast tissue with high iron levels and low antioxidant levels (reviewed in [54, 55]).

XOR is an upstream regulator of various molecules with transduction signal functions in different pathways, which may result in either pro- or antitumorigenic signaling.

In human lung microvascular endothelial cells, XOR was shown to increase the expression of the tumor suppressor protein p53, which is very often mutated and deactivated in human cancer. XOR induced oxidative stress, DNA damage, and the ROS-dependent upregulation of p53 protein, with the consequent activation of the caspase enzymatic cascade and apoptosis [17].

In 3T3-L1 murine cells, the ROS produced by the NADH-oxidizing activity of XOR were able to stimulate the activation of peroxisome proliferator-activated receptor-gamma (PPAR-*γ*), which belongs to the nuclear hormone receptor superfamily. This ligand-activated intracellular transcription factor has antiproliferative and antioncogenic activities because it can favor cell differentiation and inhibit angiogenesis [56].

XOR-derived ROS can modulate the expression of the inflammation mediator, cyclooxygenase-2 (COX-2), by either increasing or decreasing its expression. The XOR-dependent COX-2 expression in newborn mice was essential for regular kidney development, and the lack of XOR was associated with renal hypoplasia and dysplasia [57]. Additionally, XOR depletion in primary renal epithelial cells induced positive immunostaining for mesenchymal cell type markers and the lack of reactivity to E-cadherin associated with cell morphology changes from a cuboidal to myofibroblastic shape, which indicated epithelial to mesenchymal transition [58]. However, a high XOR level in human mammary epithelium lowered the COX-2 and matrix metalloprotease expression levels, which are crucial for cell migratory activity and thus for tumor progression and ability of metastasis formation [48].

XOR-generated oxidants can turn on the nuclear factor kappa-light-chain-enhancer of activated B cells (NF-*κ*B) in rat liver both during ischemia [59] and in type 1 diabetes [60]. NF-*κ*B is a transcription factor that is usually activated during chronic inflammation and in cancer, where it promotes the production of immunological cytokines and the expression of a set of antiapoptotic genes.

In U251-MG cells, derived from human brain, chemically induced hypoxia increased XOR activity and the level of XOR-derived ROS, which upregulated hypoxia-inducible factor-1 alpha (HIF-1*α*) [61]. This transcription factor is overexpressed in hypoxia and induces angiogenesis, as well as cancer invasion, thus contributing to both tumor development and progression.

## 5. Conclusions

Mammal XOR is the end product of a complicated evolutionary process leading to a hyperregulated enzyme with low specificity and highly versatile activity. In mammals, XOR has acquired many functions through the production of ROS, NO, and RNS, whereby it is involved in the triggering of key biological cell pathways and in the regulation of several physiological and pathological conditions. For these reasons, XOR represents the two faces of free radicals, which can have either negative or positive effects. XOR-derived RNS and ROS may have a cytotoxic effect. This activity may be responsible for tissue damage in hypoxia/reoxygenation and ischemia/reperfusion injury. However, this cytotoxic effect can be pharmacologically exploited to obtain selective cancerous cell killing by conjugating XOR to a specific antibody. XOR activity increases during infectious diseases and its cytotoxic action is useful for the defenses against bacteria. Additionally, XOR-derived NO and ROS have proinflammatory activity because they regulate endothelial functions, by both increasing the permeability of vascular lining and modulating the arteriolar tone. For this reason, XOR has been implicated in hypertension, cardiovascular diseases, and atherosclerosis. XOR-derived ROS are also involved in cancer pathogenesis because they may promote neoplastic transformation by activating target genes with prophlogistic, antiapoptotic, and proliferative actions. Moreover, they favor the progression to malignancy by inducing angiogenesis and cell migration. On the other hand, XOR products may activate the expression of the proapoptotic protein p53 and of transcription factors belonging to the nuclear hormone receptor superfamily with antitumorigenic and antiproliferative activity, promoting cell differentiation and inhibiting angiogenesis.

## Highlights

- XOR-derived ROS, NO, and RNS have proinflammatory and bactericidal activities.
- XOR products may be cytotoxic in many circumstances.
- XOR products modulate endothelial function and arteriolar tone.
- XOR products may induce mutagenesis, cell proliferation, and tumor progression.
- XOR products are associated with apoptosis and cell differentiation.

## Abbreviations

COX-2: Cyclooxygenase-2
FAD: Flavin adenine dinucleotide
HO^•^: Hydroxyl radical
H_2_O_2_: Hydrogen peroxide
Mo-co: Molybdenum-containing molybdopterin cofactor
NAD^+^: Oxidized nicotinamide adenine dinucleotide
NADH: Reduced nicotinamide adenine dinucleotide
NF-*κ*B: Nuclear factor kappa-light-chain-enhancer of activated B cells
NO: Nitric oxide
ONOO^−^: Peroxynitrite
RNS: Reactive nitrogen species
ROS: Reactive oxygen species
O_2_: Molecular oxygen
O_2_^•−^: Superoxide anion
XDH: Xanthine dehydrogenase
XO: Xanthine oxidase
XOR: Xanthine oxidoreductase.
